# Urine-derived stem cells in neurological diseases: current state-of-the-art and future directions

**DOI:** 10.3389/fnmol.2023.1229728

**Published:** 2023-10-30

**Authors:** Carla Cavaleiro, Gonçalo J. M. Afonso, Paulo J. Oliveira, Jorge Valero, Sandra I. Mota, Elisabete Ferreiro

**Affiliations:** ^1^CNC-Center for Neuroscience and Cell Biology, University of Coimbra, Coimbra, Portugal; ^2^Center for Innovative Biomedicine and Biotechnology, University of Coimbra, Coimbra, Portugal; ^3^Institute for Interdisciplinary Research, University of Coimbra, Doctoral Programme in Experimental Biology and Biomedicine (PDBEB), Coimbra, Portugal; ^4^Instituto de Neurociencias de Castilla y León, University of Salamanca, Salamanca, Spain; ^5^Institute of Biomedical Research of Salamanca (IBSAL), Salamanca, Spain; ^6^Department of Cell Biology and Pathology, University of Salamanca, Salamanca, Spain

**Keywords:** neurological diseases, urine-derived stem cells, advanced therapies, exosomes, neuronal differentiation, regenerative medicine

## Abstract

Stem cells have potential applications in the field of neurological diseases, as they allow for the development of new biological models. These models can improve our understanding of the underlying pathologies and facilitate the screening of new therapeutics in the context of precision medicine. Stem cells have also been applied in clinical tests to repair tissues and improve functional recovery. Nevertheless, although promising, commonly used stem cells display some limitations that curb the scope of their applications, such as the difficulty of obtention. In that regard, urine-derived cells can be reprogrammed into induced pluripotent stem cells (iPSCs). However, their obtaining can be challenging due to the low yield and complexity of the multi-phased and typically expensive differentiation protocols. As an alternative, urine-derived stem cells (UDSCs), included within the population of urine-derived cells, present a mesenchymal-like phenotype and have shown promising properties for similar purposes. Importantly, UDSCs have been differentiated into neuronal-like cells, auspicious for disease modeling, while overcoming some of the shortcomings presented by other stem cells for these purposes. Thus, this review assesses the current state and future perspectives regarding the potential of UDSCs in the ambit of neurological diseases, both for disease modeling and therapeutic applications.

## 1. Introduction

Neurological diseases encompass various categories of disorders, imposing a heavy burden on society (Thakur et al., 2016). Animal models are widely used to assess the etiopathogenesis of brain disorders and test potential treatments. However, animal models also have limitations, such as their limited genotypic variability, high cost, ethical implications, and the high complexity of the human brain, leading to different therapeutic effects (Shen, 2016; Białoń and Wąsik, 2022). *In vitro* models based on human samples are an alternative approach that can be more cost-effective than animal models. Recent advances in stem cell research have led to the use of stem cells in brain disease modeling and therapy. Stem cells have a critical advantage in that they not only provide models for understanding disease and a platform for drug discovery, but they may also have therapeutic applications on their own. Stem cells commonly used for brain disease modeling and/or therapy include embryonic stem cells (ESCs), neural stem cells (NSCs), mesenchymal stem cells (MSCs), endothelial progenitor cells (EPCs), and induced pluripotent stem cells (iPSCs). Although stem cells hold great promise for brain disease modeling and therapy, one of the biggest challenges associated with using stem cells is the high cost and low yield of the complex and multistep differentiation process required to generate brain cells, which can require invasive sample collection techniques in some cases (Fowler et al., 2020). New types of stem cells are being explored to overcome these limitations, such as urine-derived stem cells (UDSCs).

Several types of cells can be found in urine, namely epithelial cells, immune cells, and the subpopulations of UDSCs (Zhang et al., 2014; Arcolino et al., 2015; Manaph et al., 2018). UDSCs have emerged as a promising tool for stem cell research and therapy, given that they can be harvested from patients with minimal discomfort and without invasive procedures, while maintaining their genetic background (Bento et al., 2020). Importantly, UDSCs can be directly converted into other cell types, including brain cells, without the need for an intermediate conversion, which reduces costs, complexity, and risks of mutations in the process (Liu et al., 2013). Accordingly, recent data support the use of UDSCs as an *in vitro* cellular model for comprehensive precision medicine since they can allow the unveiling of patients’ specific disease biochemical mechanisms’ complexities. Moreover, they can also be used in therapy, either in their naturally occurring form or differentiated into other cell types.

In this review, we will tackle the current state of the art for UDSCs, and the future perspectives for their application in neurological diseases’ modeling and tentative therapeutic applications.

## 2. Current challenges in brain disease modeling and the emergence of stem cell research

Brain disorders represent a vast and intricate field within neuroscience, encompassing various categories of disorders, such as neurodegenerative, neurodevelopmental, cerebrovascular, infectious, tumoral, traumatic injury, and mental health disorders. Collectively, these disorders place a substantial demand on society’s capacity to overcome the economic and social burden (Thakur et al., 2016). In 2014, approximately 100 million people in the United States were affected by a neurological disorder, with an estimated cost of almost 789 trillion USD (Gooch et al., 2017). In Europe, neurological disorders were the third most common cause of premature death and disability in 2017, and their negative societal impact is expected to increase with the aging population (Deuschl et al., 2020). Therefore, finding solutions to mitigate the impact of neurological diseases is becoming increasingly crucial. The key to discovering new, translational findings is the development of effective models to gain a comprehensive understanding of diseases and design successful therapeutic approaches. However, modeling brain-associated pathologies is challenging due to the wide range of underlying causes, symptoms, and the variability of cellular and molecular effects. Hence, there are various types of models available, each with its own advantages and limitations.

Evaluating the causes of brain disorders and experimenting with potential treatments frequently makes use of animal models, which are extensively employed in research (Dawson et al., 2018). Animal models offer advantages such as the ability to study hindrances in a living, dynamic system, accounting for the functional interplay of different cell types and even organs that are not represented in cellular models. In addition, animal models allow researchers to account for various factors, including environmental or dietary alterations. These models can be created by genetically inducing brain changes specific to a given neurological disorder through germinal or neural cell-targeted mutations or pharmacologically inducing cellular degeneration using drug administration. Trauma can also be externally induced to simulate specific conditions. However, animal models have limitations. Human diseases that have complex etiologies or that are poorly understood may be misrepresented in animal models. Due to the way animal models are typically generated, diseases that do not naturally occur in animals may only be replicated by altering one of several intervening genes and overexpressing it. This may result in models that replicate the disease’s phenotype but not its underlying causes and induce the condition by mechanisms distinct from those at play in a real situation (McGraw et al., 2017). Another limitation of animal models is the limited genotypic variability of laboratory animals due to inbreeding, which can lead to a failure to represent the complexity of human genetic diversity. As a result, extrapolating results from animal models to humans may not always be successful, leading to the failure of clinical trials and the loss of considerable amounts of money, time, and lives. Even in the case of adequate representation, the high complexity of the human brain and its unique genetic and physiological dysfunctionality can lead to dissimilar therapeutic effects (Shen, 2016). In addition, animal models can be costly due to the expenses associated with acquiring and maintaining them, as well as running experiments (Białoń and Wąsik, 2022). Furthermore, modern societies are increasingly concerned about the ethical implications of using animals in scientific research. While *ex vivo* tissues from animals can also be used as a simplified model, they also require animal sacrifice.

An alternative approach to studying brain diseases involves using *in vitro* models based on human samples. While these models can also be expensive, techniques like 2D cell culture tend to be more cost-effective than animal models. While these simplified models do not account for systemic responses that may be involved in a specific disease, they do offer a more precise and focused characterization of cellular events. By using human cells, including patient-derived ones, researchers can study the disease in an autologous context. However, it’s important to note that brain cells collected in specific conditions, namely primary cultures, are often only available at more advanced stages of the disease, resulting in a poorer understanding of the disease’s earlier stages (Eichmüller and Knoblich, 2022).

In recent years, research has made remarkable progress in using stem cells for both modeling and as platforms for discovering new drugs. Additionally, stem cells have enormous potential for therapeutic applications, such as replacing damaged or dysfunctional brain cells or promoting specific biological responses, such as angiogenesis (Ryu et al., 2016). Commonly used stem cells for brain disease modeling and/or therapy include ESCs, NSCs, MSCs, EPCs, and iPSCs. Through specific differentiation protocols, these cells can be used to obtain different types of brain cells (Gage and Temple, 2013; Appaix, 2014; Terrigno et al., 2018). Despite the immense potential of stem cells, it is critical to recognize that they also come with significant limitations as models, varying in degree. One of the most significant hurdles associated with stem cell use is the high cost and low yield of the intricate and multistep differentiation process necessary for creating brain cells, which can also require invasive sample collection techniques in certain cases (Fowler et al., 2020). Additionally, incorrect differentiation into brain cells can lead to inaccurate modeling of the disease and limit their potential for therapeutic use (Strano et al., 2020). Moreover, there is a risk of tumorigenesis due to undesired mutations and potential teratoma formation in the cells generated through certain methods (Ganat et al., 2012). Another concern is the ethical considerations surrounding the use of embryonic stem cells, which require the destruction of human embryos. Despite these challenges, researchers are exploring novel types of stem cells to overcome these limitations and fully unlock the potential of stem cells for disease modeling and therapy.

Within the scope of novel disease modeling and treatment feasibility and to overcome several drawbacks, UDSCs are gaining increasing interest. They constitute a comprehensive *in vitro* cellular model and can be safely and easily isolated from patients’ urine samples. UDSCs are part of the group of body fluid-derived stem cells (BFDSCs). They offer an alternative to solid tissue-derived stem cells by also presenting characteristics of stemness, including self-renewal properties, the expression of stem cell surface markers, and multi-differentiation potential (Huang et al., 2023). BFDSCs encompasses different cell types that can be obtained from diverse sources, such as urine (Lang et al., 2013), synovial fluid (Li F. et al., 2020), menstrual fluid (Bozorgmehr et al., 2020), umbilical cord blood (Nguyen et al., 2022), peripheral blood (Pouryazdanpanah et al., 2018), breast milk (Mane et al., 2022), among others. In comparison with solid tissue-derived stem cells, BFDSCs collection is less invasive, and their isolation tends to be simpler. It bypasses the commonly used enzymatic digestion, which is otherwise applied to separate the desired cells from the extracellular matrix, using instead straightforward methods such as centrifugations to isolate the target cells fraction (Jia et al., 2018; Liu et al., 2018; Huang et al., 2023). Importantly, BFDSCs have shown significant therapeutic potential, aligning with their intrinsic biological properties. Urine sample collection is easy, fast, painless, and non-invasive, making it a safe method for obtaining UDSCs in relatively high numbers (Shi et al., 2022). Additionally, UDSCs can be obtained daily (limited by menstruation), from both healthy individuals and patients, regardless of their gender, without major ethical concerns. Furthermore, the isolation and maintenance of UDSCs are associated with significantly lower costs, adding to the appeal of using these cells. These advantages are not only applicable when compared to other sources of BFDSCs, such as those derived from breastmilk, umbilical cord blood, menstrual blood, or peripheral blood, but also when compared to alternative sources of stem cells, like adipose tissue or bone marrow mesenchymal stem cells, which may lack at least one of the aforementioned benefits. Among BFDSCs, UDSCs attributes make them an attractive option for diverse applications in precision medicine, *in vitro* pharmacological tests, regenerative medicine, and disease modeling (Bento et al., 2020).

## 3. Characterization of UDSCs

**Figure 1:**
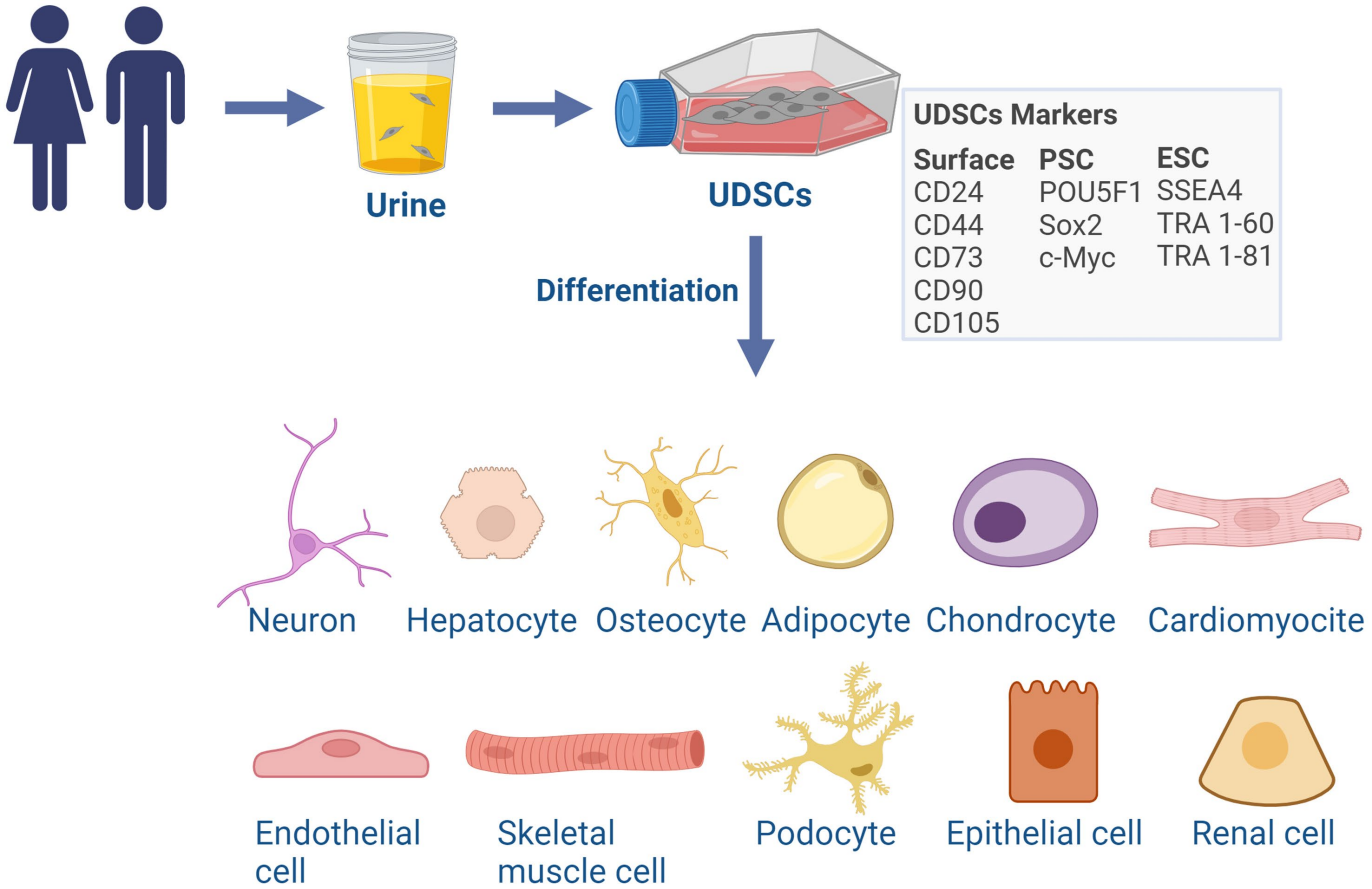
Characterization and differentiation potential of UDSCs. UDSCs can be isolated from the urine of healthy subjects or patients of all ages and have a similar phenotype to mesenchymal stroma cells (MSC). UDSCs express MSC surface markers, pluripotent and embryonic stem cell markers. UDSCs evidence a certain degree of pluripotency and can be directly differentiated into cells of the three germ layers: neurons, hepatocytes-like cells, osteocytes, adipocytes, chondrocytes, cardiomyocytes, endothelial cells and skeletal myogenic cells, renal cells, podocytes and tubular epithelial cells. ESC, embryonic stem cell; PSC, pluripotent stem cell. Created with BioRender.com.

Urine includes different cell types, broadly classified as urine-derived cells, encompassing several cellular subpopulations such as podocytes and epithelial cells from the renal tubules, nephrons, ureters, bladder, and urethra and, among others, a fraction of stem cells (Arcolino et al., 2015; Manaph et al., 2018). Those stem cells (UDSCs) represent a minor portion of urine-derived cells. UDSCs exhibit mesenchymal stromal cell (MSC)-like properties (Zhang et al., 2008) and have the ability to proliferate and to differentiate into various cell lineages (Bento et al., 2020). UDSCs express MSC surface markers including CD24, CD44, CD73, CD90, and CD105 (Dominici et al., 2006; Lv et al., 2014), the pluripotent stem cell markers POU5F1, as well as Sox2, c-Myc, among others like the ESCs markers SSEA4, TRA 1–60, TRA 1–81, common to pluripotent stem cells (Dominici et al., 2006; Manaph et al., 2018). They are also characterized by the lack of expression of the hematopoietic surface markers CD14, CD20, CD34, and CD45 (Culenova et al., 2019, 2021; Figure 1).

The biological origins and the specific mechanisms underlying the generation and shedding of UDSCs are still unclear. It was proposed that they can be biosynthesized in the proximal and distal convoluted tubules in nephrons, in the renal cortex and papilla, in the ureter, urethra, and bladder (Dörrenhaus et al., 2000; Bharadwaj et al., 2013; Bento et al., 2020). In addition, they are possibly shed into urine as part of the process of physiological tissue reparation and turnover (Dörrenhaus et al., 2000; Zhang Y. Y. et al., 2001; Zhang Y. et al., 2008).

UDSCs can be isolated from the excreted urine (non-invasive) or collected with a catheter from the upper urinary tract (invasive) of healthy subjects or patients of all ages (Zhang et al., 2008; Bharadwaj et al., 2011). Despite urine normally being a sterile fluid while stored in the body, contaminations may happen sporadically, such as through exposure to a contaminated environment at the moment of collection or in the presence of a urinary tract infection, which most frequently occurs in women (Lang et al., 2013). Additionally, still within the biological system, cells may be exposed to excretion products naturally occurring in urine such as the metabolic waste molecules ammonia, urea and creatinine, the breakdown products of drugs, among others (Bouatra et al., 2013). However, to mitigate these shortcomings, a washing step in the isolation procedure with an antimicrobial solution (Manaph et al., 2018), such as phosphate buffered saline solution enriched with antibiotics (Kim et al., 2020), may be carried out to remove possible contaminants and further help remove vestiges of urine in cell culture, along with the toxic metabolites that may be present therein.

Due to their characteristic of being plastic-adherent in the absence of coating, UDSCs can be easily separated from other urinary-derived cells in culture. During a medium change, the remaining cells are washed away, leaving behind only the adherent UDSCs in culture (Culenova et al., 2021), without the need of special substrates. Of note, a single urine collection (~70 mL) yields an average of 3 millions UDSCs which can be frozen (in passage 3, p3), and then thawed and expanded for use in experiments. In this sense, Bharadwaj and colleagues (Bharadwaj et al., 2011) evidenced that the maximum population doublings of UDSCs derived from the upper urinary tract was 56.7, corresponding to a maximum passage of p14 (Bharadwaj et al., 2011), being, in practice, used until p6 for experiments. Importantly, UDSCs maintain their proliferation, self-renewal and multi-differentiation capacity following storage at 4°C for 24 h, thus enabling their handling in batches and improving the efficacy of their processing (Lang et al., 2013). Moreover, the isolation and culture of UDSCs are cost-effective in comparison with other stem cells. UDSCs can be at least half the price and up to seven times cheaper than other stem cells regarding their obtention and maintenance, namely due to costs inherent to sample collection of the latter, which often require the need of specialized invasive medical procedures and posterior requirement of expensive substrates for cell culture (Manaph et al., 2018). Notably, these cells present a low immunogenicity (Guan et al., 2014), and absence of tumorigenicity (Zhang et al., 2008; Bharadwaj et al., 2011; Chun et al., 2012; Bharadwaj et al., 2013; Lee et al., 2013).

When in culture and still in a stem cell state, UDSCs present a “spindle,” “rice-grain” or “cobblestone” shape (Bharadwaj et al., 2011; Lang et al., 2013; Schosserer et al., 2015; Chen C. Y. et al., 2018; Wan et al., 2018). The morphology of the cells can differ slightly depending on their origin along the urinary tract. According to Chen A. J. et al., (2020) and UDSCs obtained from the renal mesenchyme have a spindle-like morphology, while cells originated in the nephron tubules are thought to be the ones with a rice-like shape. In addition, numerous studies have highlighted differences between these two morphologically distinct cell types in terms of their expression of cell surface markers, motility, proliferative capacity, and longevity in culture (Zhou et al., 2012; Chen A. J. et al., 2020; Shi and Cheung, 2021). Under *in vitro* experimental conditions, both cell types express the stemness-related markers POU5F1 and c-Myc. However, cells from renal mesenchyme origin (spindle-like cells) present enhanced motility and proliferation rate (Chen A. J. et al., 2020).

Importantly, UDSCs are characterized by a multipotential of differentiation and can be directly differentiated into cells representative of the three germ layers, such as neurons (Bharadwaj et al., 2013; Guan J. J. et al., 2014; Kang et al., 2015; Kim et al., 2018; Xu et al., 2019; Liu et al., 2020), hepatocytes-like cells (Zhou et al., 2020), osteocytes, adipocytes, chondrocytes (Guan J. J. et al., 2014), cardiomyocytes (Guan X. et al., 2014), endothelial cells (Bharadwaj et al., 2013; Kang et al., 2015) and skeletal myogenic cells (Liu et al., 2013). Moreover, UDSCs have the capacity to differentiate into renal cells, podocytes and tubular epithelial cells (Lazzeri et al., 2015), an important feature for their potential for renal repair (Figure 1). This fact suggests a degree of pluripotency, although the true extent of this characteristic is still not fully comprehended. The complete range and full extent of their biological functions are still being debated, however, their role in the promotion of tissue regeneration, replacement, and immunomodulation is already scientifically recognized (Zhang et al., 2014; Wu et al., 2021).

For all these reasons, UDSCs may be employed to perform *in vitro* pharmacological tests, in disease modeling approaches, in regenerative medicine procedures, and for precision medicine (Bento et al., 2020).

## 4. Direct conversion of UDSCs into neuronal cells

The ideal model for studying the complexity of human brain diseases should be a reliable platform that can be easily obtained from every patient, without requiring invasive procedures or raising major ethical concerns. It should be capable of producing consistent results and accurately reflecting the genetic and epigenetic background of a specific disease, all while being cost-effective and efficient. Building such a model is a huge challenge. However, certain emerging cellular-based platforms, such as UDSCs, offer promising opportunities for this purpose. Research efforts are currently underway to comprehensively understand their capabilities and limitations as a neurological model, including exploring various strategies for their direct differentiation into neuronal cells. This section presents a discussion of the relevant studies published in this area, providing details on the different protocols (Table 1) and the specific role of each compound in the differentiation media (Table 2).

**Table 1:** Protocols of UDSCs transdifferentiation into neurons.

**References**	UDSCs Passage and number of cells	Days in Culture	Media	Supplements and Growth factors	Small molecules or/and compounds
Bharadwaj et al. (2013)	Expansion	- Day 0	KSFM and EFM (1:1)	FBS (5%)	
	Passage unk.; 6.000 cells/cm^2^	Day 0	DMEM	FBS (20%), bFGF (10 ng/mL)	
		Day 1–3	DMEM		DMSO (2%), butylated hydroxyl-anisole (200 μM), insulin (5 μg/mL); human transferrin (4 μg/mL), selenium (4.2 ng/mL), KCl (25 mM), valproic acid (2 mM), forskolin (10 μM), hydrocortisone (1 μM)
Guan J. J. et al. (2014)	gelatin-coated 24-well plates		DMEM	FBS (2%), hEGF (10 ng/mL), PDGF (2 ng/mL), TGF-β (1 ng/mL), bFGF (2 ng/mL)	Cortisol (0.5 μM), adrenaline (549 ng/mL), transferrin (20 μg/mL), insulin (25 μg/mL), triiodothyronine (50 ng/mL), L-glutamine (un. conc.)
	P4, polystyrene-coated plate, 30–50% confluence	Day 0–12	DMEM/F12	B27 (2%), NEAA (non-essential amino acids, 1%), bFGF (40 ng/mL), hEGF (20 ng/mL)	L-glutamine (1%), insulin-transferrin-selenite (1%)
Kang et al. (2015)	Expansion, 100-mm culture plates		KSFM, DMEM/F12 and DMEM high glucose (2:1:1)	Penicillin (100 U/mL), streptomycin (1 mg/mL), EGF (5 ng/mL), BPE (50 ng/mL)	Cholera toxin (30 ng/mL)
	Passage unk.; 1 × 10^5^ cells/well in 24-well plates, 80% confluence	Day 0–7	NeuroCult differentiation Kit		
Kim et al. (2018)	Expansion		KSFM, DMEM (high glucose) and DMEM/F12 (2:1:1)	FBS (5%), PS (1%), BPE (25 ng/mL), EGF (7.5 ng/mL)	Hydrocortisone (0.2 ng/mL), insulin (2.5 ng/mL), transferrin (2.5 μg/mL), triiodothyronine (100 μM), adenine (90 μM), cholera toxin (19.2 ng/mL)
	P4, 80% confluence; laminin-coated dishes (5 μg/cm^2^)	Day 0–14	DMEM/F12	B27 (2%), nonessential amino acids (1%), PDGF-BB (5 ng/mL)	L-glutamine (1%), and retinoic acid (10 mM)
Xu et al. (2019)	Expansion		SingleQuot Kit CC-4127 Renal Epithelial Cell Growth Medium (REGM), DMEM/F12	FBS (10%), NEAA (0.1 mM), GlutaMAX (1 mM), and PS (unk. Conc.)	
	P4-P5, matrigel-coated (1%) 24-well plates (20000–30,000 cells/well)	- Day 2	SingleQuot Kit CC-4127 Renal Epithelial Cell Growth Medium (REGM), DMEM/F12	FBS (10%), NEAA (0.1 mM), GlutaMAX (1 mM), PS (un. conc.)	
		Day 0–7	DMEM/F12	EGF (50 ng/mL), FGF10 (100 ng/mL)	Nicotinamide (0.5 mM), Y27632 (10 μM), A8301 (5 μM), CHIR99021 (3 μM), TTNPB (1 μM), forskolin (5 μM); valproic acid (0.5 mM), sodium butyrate (0.1 mM)
		Day 7–17	DMEM/F12 and Neurobasal medium (1:1)	N2 (0.5%), B27 (1%), PS (unk. Conc.), bFGF (20 ng/mL), BDNF (20 ng/mL), GDNF (20 ng/mL), NT3 (20 ng/mL)	cAMP (100 μM), Y27632 (10 μM), A8301 (5 μM), CHIR99021 (3 μM), TTNPB (1 μM), forskolin (5 μM), vitamin C (0.2 mM)
			DMEM/F12 and Neurobasal medium (1:1)	N2 (0.5%), B27 (1%), PS (unk. Conc.), BDNF (20 ng/mL), GDNF (20 ng/mL), NT3 (20 ng/mL)	
Liu et al. (2020)	Isolation to colonies	3 days	DMEM/F12	FBS (10%), PS (1%), Renal Epithelial Cell Growth BulletKit (0.1%)	
		~ Days	Renal Epithelial Basal Medium (REBM)	FBS (1%), PS (1%), Renal Epithelial Cell Growth BulletKit (0.1%)	
	Expansion	~ Days	KSFM: DMEM/F12 (1:1)	FBS (10%), PS (1%), BPE (50 μg/mL), EGF (10 ng/mL)	Adenine (1.8 × 10^−4^ M)
	P3. matrigel-coated wells, 1 × 10^4^ cells/cm^2^	Day 0	Neurobasal medium	B27 (2%), N2 (1%), PS (1%)	L-glutamine (1%), cAMP-Na (100 μM), VPA (4 μM), CHIR99021 (3 μM), Repsox (1 μMr), Forskolin (10 μM), SP600125 (10 μM), GO6983 (5 μM), Y-27632 (10 μM), IBET151 (2 μM), Isoxazole 9 (20 μM), retinoic acid (10 μM), QVD-OPh (5 μM), vitamin C (0.2 mM)
		Day 3	Neurobasal medium	B27 (2%), N2 (1%), PS (1%), with BDNF (20 ng/mL), GDNF (20 ng/mL), IGF (20 ng/mL), NT3 (20 ng/mL)	L-glutamine (1%), cAMP-Na (100 μM), VPA (4 μM), CHIR99021 (3 μM), Repsox (1 μMr), Forskolin (10 μM), SP600125 (10 μM), GO6983 (5 μM), Y-27632 (10 μM), IBET151 (2 μM), Isoxazole 9 (20 μM), retinoic acid (10 μM), QVD-OPh (5 μM), vitamin C (0.2 mM)
		Day 11–14	Neurobasal medium	B27 (2%), N2 (1%), PS (1%), BDNF (20 ng/mL), GDNF (20 ng/mL), IGF (20 ng/mL), NT3 (20 ng/mL)	L-glutamine (1%)

BDNF, brain-derived neurotrophic factor; bFGF, basic fibroblast growth factor; BPE, brain pituitary extract; conc, concentration; DMEM, Dulbecco’s Modified Eagle Medium; DMSO, dimethyl sulfoxide; EFM, embryonic fibroblast medium; EGF, epidermal growth factor; FBS, fetal bovine serum; FGF10, Fibroblast Growth Factor 10; KSFM, keratinocyte serum-free medium; hEGF, human epidermal growth factor; NT3, neurotrophin-3; PDGF, platelet-derived growth factor; PS, penicillin/streptomycin; TGF-β, transforming growth factor beta; unk., unknown.

**Table 2:** Core compounds used among the existing protocols for the differentiation of UDSCs into neuron-like cells and main functions.

Compound	Biological effect
A8301	Inhibitor of the TGF-β pathway (Tojo et al., 2005), involved in cell growth, differentiation, development, immune response, tissue homeostasis and the determination of the neural fate (Wang et al., 2016)
BDNF	Neurotrophic factor. Promotes the differentiation of NPCs into specific neuronal lineages and neuronal maturation (Bartkowska et al., 2007; Colucci-D’amato et al., 2020)
bFGF	Promotor of growth, survival, and regulation of neurogenesis (Li et al., 2022)
BHA	Antioxidant used to prevent cell damage due to oxidative stress during the induction process (Delanghe et al., 2021)
cAMP	Intracellular signaling molecule. Induces neuronal differentiation, maturation, and survival (Hansen et al., 2000; Li et al., 2000; Lepski et al., 2013)
CHIR99021	Inhibitor of GSK3β, whose inactivation leads to the activation of the canonical Wnt signaling pathway, which plays a key role in neural development and differentiation (Huang et al., 2017)
EGF	Promotor of cell expansion, survival and differentiation (Li et al., 2022)
FGF10	Promotor of neurogenesis initiation; regulator of cell proliferation (Sahara and O’Leary, 2009; Guillemot and Zimmer, 2011)
Forskolin	Activator of adenylate cyclase (Alasbahi and Melzig, 2012), promoting gene expression modulation and neuronal differentiation (Thompson et al., 2019)
GDNF	Neurotrophic factor inducing NPCs differentiation into specific neuronal lineages, damaged neurons regeneration, and neuronal maturation (Cortés et al., 2017)
GO6983	Inhibitor of PKC, related to the modulation of stem cell pluripotency (Rajendran et al., 2013)
Hydrocortisone	Modulator of neurogenesis, neuronal maturation (Aden et al., 2011; Odaka et al., 2017)
IBET151	Inhibitor of proteins of the BET family, related to the regulation of gene expression, improving the rate of reprogramming (Li et al., 2015; Wu et al., 2015)
IGF	Growth factor, involved in regulation of neurogenesis and synaptogenesis (Nieto-Estévez et al., 2016)
Insulin	Inductor of a metabolism prone for cell growth and development
Isoxazole9	GSK-3β inhibitor (Sayed et al., 2023). Trigger of calcium influxes (Koh et al., 2015). These lead to the promotion of the transcription of genes involved in neuronal differentiation and maturation
L-Glutamine	Promotor of cell survival and growth. Precursor for the synthesis of glutamate (Bak et al., 2006; Yoo et al., 2020)
Laminin	Substrate for cell migration and attachment, promoter of neurite outgrowth and axonal guidance (Mruthyunjaya et al., 2010)
NaB	Inhibitor of histone deacetylase which facilitate gene expression, promoting neuronal differentiation (Jaworska et al., 2019) and attenuation neuronal apoptosis (Zhou et al., 2021)
Nicotinamide	Inductor of the expression of neuronal markers, neuroprotector, selective kinase inhibitor, and suppressor of the expression of meso-endoderm markers (Meng et al., 2018)
NT-3	Neurotrophic factor, Wnt/β-catenin signaling pathway modulator. Promotes neuronal differentiation of NPCs (Yan et al., 2021)
PDGF	Neurotrophic factor and promotor of cell survival involved in NPCs differentiation (Funa and Sasahara, 2014)
QVD-OPh	Inhibitor of caspases, anti-apoptotic (Kuželová et al., 2011)
Repsox	TGF-β pathway inhibitor. Increases neuroepithelial markers levels and promotes neuronal differentiation (Li and Huang, 2019)
Retinoic acid	Metabolic product of vitamin A involved in NPCs differentiation into specific types of neurons (Maden, 2007)
SP600125	Inhibitor of the c-Jun N-terminal kinase (JNK) signaling pathway. This leads to a reduction in stemness and promotes cellular differentiation (Semba et al., 2020)
Selenium	Oxidative stress and other damaging factors protector (Van et al., 2003)
Transferrin	Neuroprotector, part of the antioxidant defense system, iron metabolism (Van et al., 2003)
TTNPB	Agonist of RA receptor subtype important for ensuring stem cell reprogramming into neuronal-like cells (Das and Pethe, 2021)
Valproic Acid	Inhibitor of histone deacetylase, leading to changes in chromatin structure and gene expression that favor neuronal differentiation (Phiel et al., 2001; Vukićević et al., 2015)
Vitamin C	Neuroprotective effects, antioxidant (May, 2012), enhancer of the expression of genes involved in neurogenesis, maturation, and neurotransmission (Shin et al., 2004)
Y27632	Rho-kinase inhibitor, which indirectly inhibits the ERK signaling pathway promoting stem cells survival and differentiation (Kamishibahara et al., 2016; Fu et al., 2018)

Several scientific reports have demonstrated the successful direct conversion of UDSCs into various cell types across different germline lineages. For instance, hepatocyte-like cells have been achieved (Zhou et al., 2020), along with the differentiation into osteocytes, adipocytes, and chondrocytes (Guan J. J. et al., 2014). Furthermore, different authors have accomplished the direct conversion of UDSCs into neuron-like cells using various approaches, resulting in cells with distinct characteristics (Bharadwaj et al., 2013; Guan J. J. et al., 2014; Kang et al., 2015; Kim et al., 2018; Xu et al., 2019; Liu et al., 2020).

In 2013, Bharadwaj and colleagues (Bharadwaj et al., 2013) used a multistep protocol to achieve the differentiation of UDSCs into neuron-like cells. At D3 (maximum days in culture), cells had already undergone morphological changes, including the emergence of extensions and processes. Forty % of cells in culture were expressing nestin, and NF200 (neurofilament 200), characteristic markers of neural progenitor cells (NPCs) and neuronal cells, respectively. This differentiation success rate indicates that there was still room for efficiency enhancement of the neuronal differentiation process, along with improved neuronal maturation (Bharadwaj et al., 2013).

The following year, another study achieved the transformation of UDSCs into NPCs (Guan J. J. et al., 2014). At D12, cells differentiated from UDSCs were expressing the Sox2 and Nestin (markers of NPCs) in higher levels than NSE (neuron-specific enolase) and β-III-tubulin, both neuronal markers. It is relevant to highlight that at D12, the maximum number of days that the authors maintained cells in culture, β-III-tubulin expression was residual, indicating that the obtained neuron-like cells were in an early stage of neuronal differentiation. Additionally, in this study, UDSCs were transplanted into a rat brain, being able to survive and migrate while expressing proteins characteristic of a neuronal phenotype, demonstrating the potential of UDSCs to differentiate into neuronal-like cells in the brain.

In another study (Kang et al., 2015), the capacity of UDSCs to differentiate into neuron-like cells was compared to the one from adipose tissue stem cells (ADSCs). The authors found that UDSCs have a better neurogenic differentiation rate than ADSCs, suggesting that these cells are more suitable to be converted into neurons (Kang et al., 2015).

Later, in 2018, Kim and colleagues (Kim et al., 2018) optimized a protocol to obtain mature neurons from UDSCs recurring to laminin coating and/or treatment with platelet-derived growth factor BB (PDGF-BB). After 14 days of neuronal induction, the authors observed an increase in the expression of early neuronal differentiation markers (nestin and β-III-tubulin) and mature neuronal cells [NeuN (hexaribonucleotide binding protein-3 or Fox-3), MAP2 (microtubule-associated protein 2), and NF-M (neurofilament marker)] in the PDGF-BB treated UDSCs cultured on laminin-coated plates. This suggests a synergistic effect between laminin and PDGF-BB to promote a higher degree of neuronal maturity.

A more recent investigation (Xu et al., 2019) pursued the differentiation of UDSCs into neuron-like cells through a distinct methodology. The study employed a direct reprogramming two-step neuronal induction approach, utilizing seven small molecules and growth factors. By establishing this protocol the authors were able to obtain a higher degree of neuronal maturation and conversion efficiency when compared to the previous reports. The alteration of the UDSCs morphology into a more neuron-like morphology was observed early in the application of the protocol, being visible after 24 h of NIM application. At D5, 58% of cells expressed β-III-tubulin. At D17, cells depicted a more extended neurite outgrowth with dendrite-like structures and apart from β-III-tubulin, also expressed MAP2, NeuN, Tau, SYN (synapsin), and NF-01, being negative to GFAP (glial fibrillary acidic protein). The expression of these neuronal markers and neuronal morphology was maintained through D30. At D17, the authors further characterized the neuronal type generated using this protocol and confirmed its glutamatergic nature, as evidenced by the expression of the glutamatergic marker glutamate. Additionally, markers associated with other neuron types, such as GABA (gamma-aminobutyric acid), HB9 (homeobox HB9), and TH (tyrosine hydroxylase), were rarely detected. To assess the functionality of the neurons derived from UDSCs, whole-cell patch-clamp recording was employed. Following the complete differentiation process, cells at D17 exhibited partial electrophysiological properties, displaying both outward and inward currents. However, neither at D17 nor at D47 were the cells able to generate action potentials. The study conducted by Xu and colleagues (Xu et al., 2019) represented a significant advancement in establishing a neuronal differentiation protocol for converting UDSCs. However, the successful conversion of UDSC into functional neurons has not yet been accomplished, underscoring the pressing requirement for additional research in this field.

Another differentiation approach, also recurring to small molecules, was performed by Liu and collaborators (Liu et al., 2020), who obtained neuron-like cells, with a discernible subset exhibiting GABAergic characteristics. By D6 cells were expressing the neuronal markers Tuj-1, MAP2, Tau, and PSA-Ncam (polysialylated neuronal cell adhesion molecule). At D14, a small subset of cells exhibited GABA positivity, indicative of GABAergic neurons. There was also no evidence of ChAT- or TH-positive cells (cholinergic or dopaminergic neurons, respectively), despite the majority of cells expressing typical neuron-specific genes such as *Tau, MAP2, Ncam*, and *NeuN*. The authors did not assess glutamate expression, unlike in the previous study, making it difficult to comprehend the similarities and differences between the neuron-like cells generated using these two protocols. Furthermore, the resultant cells displayed voltage-gated channels for Na^+^ and K^+^, characteristic of excitable cells, but lacked Ca^+^ currents, thereby failing to generate action potentials. Based on these findings, it can be concluded that the conversion of UDSCs in both studies (Xu et al., 2019; Liu et al., 2020) did not result in fully matured neurons.

**Figure 2:**
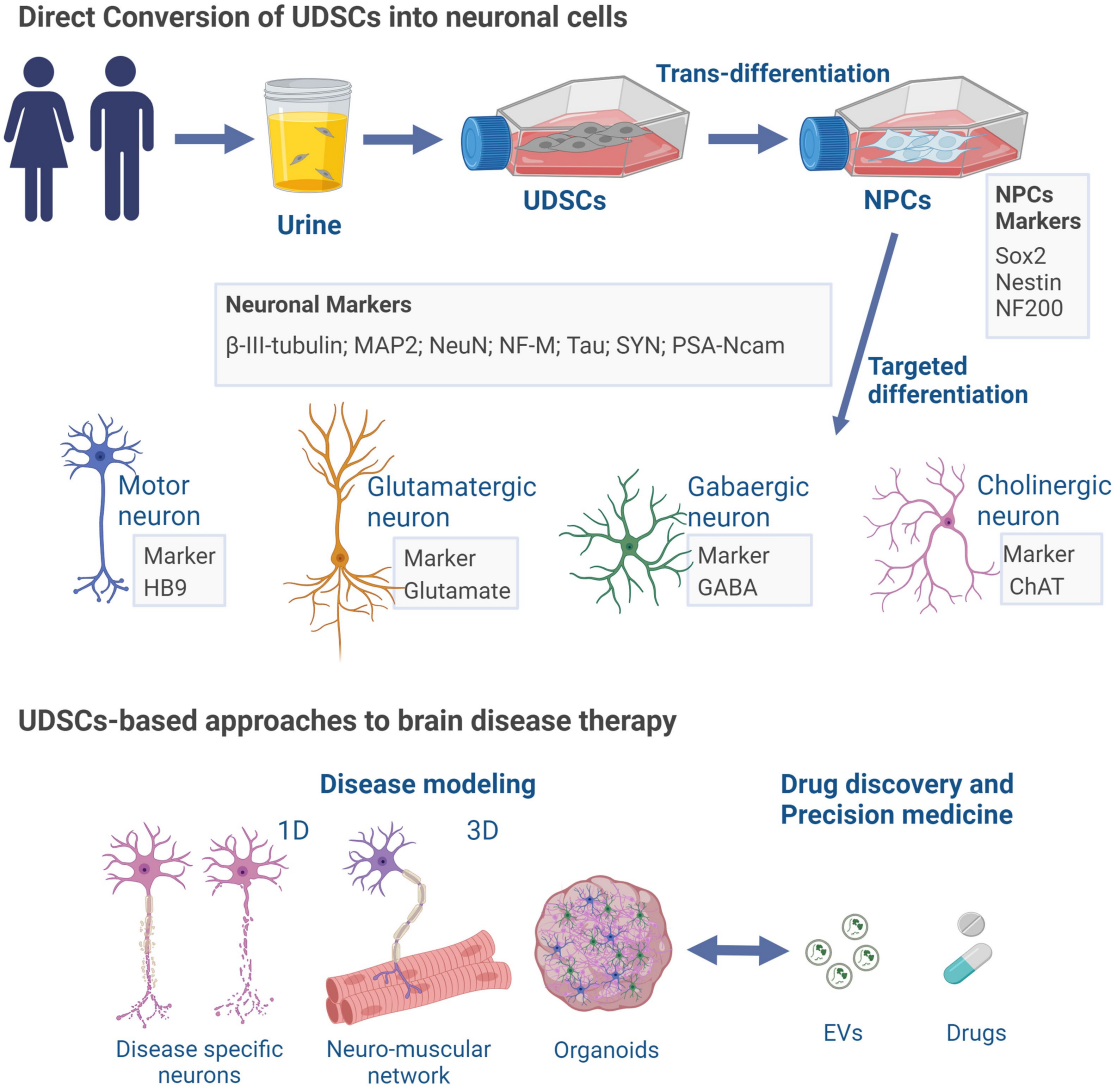
UDSCs-induced neuronal differentiation and brain disease applications. UDSCs isolated from the urine can be directly converted into neuron-like cells divided into different neuronal type depending on their specific markers (motor, glutamatergic, gabaergic or cholinergic). UDSCs-derived neurons may be employed in disease modeling approaches, to perform *in vitro* pharmacological tests, and for precision medicine. ChAT, Choline acetyltransferase; EVs, extracellular vesicles; GABA, gamma-aminobutyric acid; HB9 homeobox HB9; MAP2, microtubule-associated protein 2; NeuN, hexaribonucleotide binding protein-3; NFM, neurofilament marker; NF200, neurofilament 200; NPCs, neural progenitor cells; PSA-Ncam (polysialylated neuronal cell adhesion molecule); SYN, synapsin. Created with BioRender.com.

Similar to the potential of UDSCs to differentiate into neuron-like cells, other cells found in urine hold valuable clinical applications as an important source for the conversion into neuronal cells and disease modeling. In line with this, various studies have reported the generation of disease-related neuronal cells through the reprogramming of urine cells. These methodologies aim to achieve neuronal cells through direct differentiation, or through urine cells reprograming into iPSCs for further neuronal differentiation; and involved the use of retroviruses (Zhang et al., 2016), episomal vectors (Liu et al., 2020), or a reprogramming medium supplemented with small molecules (Yi et al., 2018). Thus, small molecules can be used to differentiate urine-derived renal cells into neuron-like cells showing neuron-specific genes expression and voltage gated Na^+^ and K^+^ currents (Liu et al., 2020). Similarly, Zhang and colleagues were able to obtain induced-neurons expressing multiple neuron-specific proteins and capable of generating action potentials from urine cells from control individuals and Wilson’s disease patient, through the overexpression of the transcription factors Ascl1, Brn2, NeuroD, c-Myc, and Myt1l (Zhang et al., 2016). Importantly, iPSCs obtained from urine sample can be used to generate extended pluripotent stem cells (EPSCs) which could then be differentiated to neuronal progenitor cells (Hao et al., 2023). Moreover, urine-derived iPSCs previously reprogramed can also be differentiated into functional motor neurons capable of forming neuromuscular junctions in co-cultures with muscle cells (Yi et al., 2018). In the same line, urine-derived iPSCs from Down Syndrome individuals have been used to obtain cells differentiated into glutamatergic neurons (Lee et al., 2017). Furthermore, in a recent breakthrough, Teles e Silva and colleagues demonstrated the pioneering use of urine-derived iPSCs in producing human cerebral organoids to model Down Syndrome for the first time (Silva AL et al., 2023). Of note, these cerebral organoids faithfully replicate early features of human cortical development, encompassing the organization of neural progenitor zones, programmed differentiation of both excitatory and inhibitory neurons, and the presence of upper and deep-layer cortical neurons, along with astrocytes (Silva AL et al., 2023). Important hallmarks have been achieved, namely the obtention of mature neurons or neurons carrying the phenotype of a specific diseases. As such, studies of this nature might provide some insights that could prove useful in guiding future works that aim to achieve disease models of neurological diseases based on UDSCs due to the close nature between them (Figure 2).

## 5. UDSCs-based approaches to brain disease therapy

### 5.1. UDSCs *in vivo* transplantation

The therapeutic potential of UDSCs for neurological diseases remains relatively unexplored, yet the limited findings obtained thus far hold promise. UDSCs engraftment or their secretomes have shown to be effective mostly in preclinical reports of renal and bladder diseases, such as acute kidney injury (Li X. et al., 2020), renal acute tubular injury (Grange et al., 2020) or bladder fibrosis (Wu et al., 2023), evidencing a consistent tissue (Li F. et al., 2020; Li X. et al., 2020) regeneration effect, mainly through their pro-angiogenic, anti-apoptotic and anti-inflammatory effects (Grange et al., 2020; Li F. et al., 2020; Li X. et al., 2020 Wu et al., 2023), over the living afflicted tissues. Within the field of neurological disorders, very few studies of UDSCs *in vivo* preclinical engraftment have been performed. Nevertheless, in the ones available in the literature, transplanted UDSCs have been shown to have multiple differentiation potential, chemotaxis ability, and an undeniable functional recovery and cellular neuroprotective effect. In more detail, UDSCs, when embedded in hydrogel scaffolds and transplanted into rat’s craniotomy-lesioned motor cortex, survive at least 3 weeks and present full *in vivo* differentiation potential into astrocyte resembling morphology cells, expressing neurogenesis-related markers such as GFAP, β-III-tubulin, and nestin. Importantly, transplanted cells were found not only surrounding the lesion site, but also functionally integrating hippocampus neural tissue, thus demonstrating migration abilities (Guan J. J. et al., 2014). In the context of spinal cord injury (SCI), in comparison with the control group, the transplant of UDSCs promoted motor functional recovery in a rat model of SCI (Chen H. et al., 2018). Additionally, the authors showed that the injection of UDSCs in conjugation with chondroitinase ABC, an enzyme involved in axon regeneration (Day et al., 2020), generated a synergistic effect on faster motor function recovery of the lesioned animals (Chen H. et al., 2018). Later, Muir and collaborators evidenced that functional recovery observed in the hUDSCs+ ChABC animals engrafted group was related to the increase mRNA expression levels of BDNF and nerve growth factor (NGF), both neuroprotective neurotrophins (Muir et al., 2017), and previously described as involved in post-lesion tissue repair (Hasan et al., 2017).

Importantly, UDSCs have a significant advantage over other stem cells, such as the absence of undesired immune response (le Blanc et al., 2003; Chun et al., 2012; Guan et al., 2015; Kang et al., 2015; Wu et al., 2018; Bento et al., 2020) and teratoma formation (Zhang et al., 2008; Bharadwaj et al., 2011; Chun et al., 2012; Bharadwaj et al., 2013; Lee et al., 2013). In particular, UDSCs, do not express HLA-DR glycoproteins (le Blanc et al., 2003; Guan et al., 2015; Kang et al., 2015; Chen L. et al. 2018; Wu et al., 2018; Bento et al., 2020) until around p7 *in vitro*, often responsible for triggering transplant rejection, as happening with hematopoietic (Gabbianelli et al., 1987; Park and Seo, 2012) and adipose stem cells (Dam et al., 2021). In addition, UDSCs isolated from healthy subjects, which retained a normal karyotype *in vitro*, did not induce an adverse response and scaffold rejection after transplantation into an animal model of traumatic brain injury (Guan J. J. et al., 2014).

By bridging the bench with the bedside, animal models provided direct evidence of the feasibility of transplanted stem cells’ regenerative properties, therefore, anticipating the efficacy and safety of specific clinical procedures. The existent studies using animal models and grafting strategies emphasize the necessity of exploring the therapeutic potential of UDSCs and the underlying cellular and molecular mechanisms in different neuropathological contexts.

### 5.2. UDSCs secretome-based approaches to brain diseases therapy

Similarly, to the therapeutic effects in the scope of direct UDSCs transplantation, preclinical studies employing UDSCs-derived secretome also show promising results for the treatment of brain disorders. UDSCs-related secretomes’ regenerative and immunomodulatory properties arose in several diseases, from urological (Grange et al., 2020; Li X. et al., 2020; Wu et al., 2023) to diabetic wound healing (Zhao et al., 2018). Several authors hypothesized that UDSCs therapeutic may depend on these cells’ ability to secrete various bioactive cargoes. Among them, we can highlight miRNAs, such as miR-26a (Ling et al., 2020), related to neurogenesis enhancement; miR-21-5p (Nasci et al., 2019) and miR-26a-5p (Wan et al., 2022), both involved in cell proliferation; growth factors such as VEGF (Jiang et al., 2016), IGF-1 and EGF (Zhu et al., 2018); DMBT1 and TIMP1 proteins (Chen C. Y. et al., 2020), angiogenin (Jiang et al., 2016), Klotho protein (Grange et al., 2020) and matrilin-3 (MATN3) (Zidan et al., 2021). Importantly, these biomolecules act as paracrine factors over recipient cells (He et al., 2018), exerting regulatory effects in several cellular mechanisms, including proliferation, differentiation, cell fate and survival, neuroprotection, neurogenesis, angiogenesis, and tissue regeneration. A great amount of these studies encompass the use of exosomes, a group of cup-shaped extracellular vesicles (EVs) (Guo et al., 2021), identifiable in biofluids and that shuttle multiple bioactive cargos, able to influence recipient cells. Therefore, we will focus in this section on the therapeutic use of UDSCs- derived EVs.

UDSCs have been also associated with the release of heterogeneous EVs, ranging from microvesicles (150 nm to 1 μm) to exosomes (60 to 150 nm) (Zidan et al., 2021). Importantly, like in the case of other EVs, the content of UDSCs-derived EVs often reflects their origin and the pathophysiological environment in which they were generated (Arcolino et al., 2015). Additionally, EVs from healthy UDSCs may be used for therapeutic purposes.

Anti-inflammatory properties of UDSCs-secreted EVs have been assessed, opening new possibilities for their therapeutic use in immunodeficiency scenarios (Zidan et al., 2021). Particularly, UDSCs-derived exosomes have been shown to contain immunomodulatory molecules such as the cytokines TGF-β1 (Jiang et al., 2016), B-cell activating factor (BAFF), A proliferation-inducing ligand (APRIL), interleukin-6 (IL–6), and the CD40 ligand protein (CD40L), which stimulate B cells to initiate the immune response without T cell proliferation stimulation, possibly due to the presence of immunosuppressive cytokines and miRNAs, such as miRNA146–5p (Zidan et al., 2021). Furthermore, miR-146a-5p has been identified to target the interleukin-1 receptor-associated kinase 1 (IRAK1) mRNA, which degradation results in nuclear factor (NF)-κB signaling inhibition (Li X. et al., 2020). Downregulation of IRAK1 may also be involved in the modulation of the phosphoinositide 3-kinase (PI3K)/Akt and the mitogen-activated protein kinase (MAPK) pathways, leading to a decrease in apoptosis and inflammatory cytokines in podocytes (Zhang et al., 2018).

Although studies are scarce, some articles demonstrate the benefits of using EVs from UDSCs, most notoriously exosomes, to improve some brain-related disorders. For instance, in a rat model of ischemic stroke, direct intravenous injection of exosomes derived from human UDSCs, 4 h after stroke induction, resulted in a reduction of infarct volume and enhancement of the subventricular zone (SVZ) endogenous neurogenesis, via miR-26a/HDAC6 signaling pathway activation, suggesting the neuroprotective effect of those EVs (Ling et al., 2020). Moreover, in a mouse model of Rett Syndrome, administration of UDSCs-derived exosomes improved the motor coordination, cognition, and behavioral symptoms presented by the animals (Pan et al., 2021). Importantly, this effect was related to the enhancement of neurogenesis in the SVZ, resulting from the integration of EVs enriched in miR-21-5p by the neural stem cells. miR-21-5p, a regulator of cell proliferation, mitochondrial respiration, and angiogenesis (Nasci et al., 2019) further inhibited the EPha4/TEK axis, resulting in neurogenesis enhancement, as evidenced by the increased expression of β-III-tubulin and DCX (doublecortin) (Pan et al., 2021). Following spinal cord injury, UDSCs-derived exosomes induce motor neurons functional recovery, concomitantly with local endothelial cells microvessel regeneration through ANGPTL3 (Angiopoietin-like 3)-mediated PI3K/AKT signaling pathway activation (Cao et al., 2021), which promotes angiogenesis process (Jiang et al., 2019). Additionally, Dan and colleagues recently suggested that UDSCs-derived exosomes prevented apoptosis of aging retinal ganglion cells (RGCs) (Dan et al., 2023). The authors exposed aging RGCs to UDSCs conditioned medium and observed the promotion of cell survival and proliferation (Dan et al., 2023). However, since the authors did not analyze the presence of exosomes in the culture medium used, it is not possible to assure that those were the mediators in the effects observed.

Overall, UDSCs-related therapies, whether they depend on a cell transplant or cell-derived secretome treatment, have shown promising results, both leading to tissue repair. Effects depend mainly on the activation of regeneration-associated signaling pathways, along with immunomodulatory properties, showing promise within this scope for future studies.

## 6. Future perspectives

UDSCs can be safely and easily isolated from patients’ urine samples, expanded *in vitro*, and differentiated into different cell types, including neuron-like cells (Bharadwaj et al., 2013; Guan J. J. et al., 2014; Kang et al., 2015; Xu et al., 2019; Liu et al., 2020). However, they still present some limitations due to difficult culturing conditions such as exposition to a wide range of contaminants (e.g., infections), as well as metabolic waste products, which can potentially affect them. Some of these disadvantages should be overcome by improving the current methodological procedures or developing new ones.

Despite being in its nascent stages, the study of UDSCs elicits optimism, primarily rooted in promising preclinical research showcasing their biocompatibility, engraftment, and *in vivo* differentiation potential. As stated in section 5, preclinical studies evaluating the therapeutic properties of UDSCs have been exclusively conducted in small animal models. Thus, further investigations are imperative to scrutinize UDSCs post-injection reprogramming prowess and functional efficacy.

Patient UDSCs, and specifically their derived EVs, can be engineered to carry specific cargo that will exert a neuroprotective activity over targeted cells or, the attenuation of disease-related dysfunctional signaling pathways (Drago et al., 2013). The direct delivery of these cargo and its amplification through the control induced-expression of surface antibodies, to be recognized by specific cell types (Bryniarski et al., 2013) renders a new field to be exploited.

## 7. Conclusion

UDSCs are notorious for their ease, non-invasive process of obtention, able to be simply isolated from any patient’s urine, without major ethical concerns, which makes them a promising alternative to other cell types that can be used for the modeling of neurological disorders and theranostics. Preclinical reports provide evidence of the therapeutic and modeling potential of these cells and their secretome for a multitude of central nervous system-related disorders. UDSCs may be directly reprogrammed into the desired cell type, without the need for pluripotency induction as for iPSCs. Importantly, UDSCs may differentiate *in vitro* and *in vivo* into neuronal cells and evidence immunomodulatory and neuroreparative properties. Thus, UDSCs hold great promise as a research field for the development of new brain therapies that could enhance the quality of life for patients with neurological diseases.

## Author contributions

GA, CC, SM, and EF have reviewed the literature and wrote the first draft of the manuscript. PO, JV, SM, and EF edited and revised the manuscript. All authors contributed to manuscript revision, read, and approved the submitted version.

## Abbreviations

ADSCs: Adipose tissue stem cells
BDNF: Brain-derived neurotrophic factor
bFGF: Basic fibroblast growth factor
DMEM: Dulbecco’s Modified Eagle Medium
EGF: Epidermal growth factor
EPCs: Endothelial progenitor cells
ESCs: Embryonic stem cells
EVs: Extracellular vesicles
FBS: Fetal bovine serum
FGF10: Fibroblast growth factor 10
GDNF: Glial cell-derived neurotrophic factor
GFAP: Glial fibrillary acidic protein
iPSCs: Induced pluripotent stem cells
KSFM: Keratinocyte Serum-Free Medium
MSCs: Mesenchymal stem cells
NEAA: Non-essential amino acids
NIM: Neuronal Induction Medium
NM: Neuronal Medium
NMM: Neuronal Maturation Media
NPCs: Neural progenitor cells
NSCs: Neural stem cells
RA: Retinoic acid
REGM: Renal Epithelial Cell Growth Medium
RGCs: Retinal ganglion cells
SVZ: Subventricular zone
UDSCs: Urine-derived stem cells
VPA: Valproic acid
